# Effects of Xinfeng Capsules on Expression of Platelet Granule Membrane Protein 140 and Platelet Cluster of Differentiation 40 Ligand in Peripheral Blood of Adjuvant Arthritis Rats

**DOI:** 10.1155/2012/139696

**Published:** 2012-05-07

**Authors:** Zong Rui-kai, Liu Jian

**Affiliations:** ^1^Hubei University of Traditional Chinese Medicine, Hubei, Wuhan 430065, China; ^2^Department of Rheumatism and Immunity, The First Hospital Affiliated to Anhui College of Traditional Chinese Medicine, Anhui, Hefei 230031, China

## Abstract

Platelet GMP-140 and CD40L as specific markers of platelet activation play an important role in the morbidity and development of rheumatoid arthritis. The expression of GMP-140, CD40L increases in peripheral blood of AA rats. And they have correlation with voix pedis' swelling, AI. XFC could inhibit the inflammatory response through inhibiting platelet activation of AA rats, which means decreasing the expression of GMP-140, CD40L in peripheral blood. So, the voix pedis' swelling and AI were decreased as the result.

## 1. Introduction

Studies show that platelet activation plays an important role in the pathogenesis of rheumatoid arthritis [1–3]. Activation of platelet releases and expresses a variety of activation products to the membrane surface. These substances participate in platelet-endothelial cell reaction and platelet-leukocyte reaction in different ways and play an important role in the occurrence and development of inflammation and particularly, chronic inflammatory [4]. To explore the mechanism of platelet activation in the pathogenesis of rheumatoid arthritis, the rats were injected with Freund's complete adjuvant (FCA) as immune stimulation, to observe the change of platelet GMP-140 and CD40L in peripheral blood and effects of Xinfeng Capsules (XFC) on them. The result of study is as follows.

## 2. Material and Methods

### 2.1. Animals

40 male clean degree SD rats, Anli laboratory animals limited liability company of Anhui Province (certificate number: 2010-0008), average weight (200 ± 20) g.

### 2.2. Major Drugs and Reagents

Methotrexate (MTX): 2.5 mg per tablet, Sine Pharmaceutical factory of Shanghai pharmaceutical limited, batch number: 100923; tripterygium wilfordii polycoride Tablet (TPT): 10 mg per tablet, Shanghai Fudan Fuhua pharmaceutical industry limited, batch number: 100809. XFC: preparation centre of first hospital affiliated to Anhui College of Traditional Chinese Medicine, main composition: coix seed, Astragalus, centipede, tripterygium wilfordii, 0.4 g per pill, batch number: 100521; FCA: USA Sigma company, batch number: 065K7805; anti-rat GMP-140 and CD40L monoclonal antibody labeled with Fluorescein Isothiocyanate (FITC): Beijing Bioss biotechnology Co. Ltd, batch number: 100512 and 100419, Art. No. bs-1286R and bs-0561R.

### 2.3. Major Instruments and Equipments

Beckman-Coulter XL-ESPIC MCL-type Flow Cytometry.

### 2.4. Model and Administration

40 SD male rats were randomly divided into 5 groups after adaptable feeding 1 week, normal control group (*n* = 8), and model groups (*n* = 32). Except for the rats of normal group, the others were intracutaneously injected with 0.1 mL of Freund's complete adjuvant in the right hindlimb to model [5]. AA rats were randomly divided into 4 groups and administrated at 20 times clinical dosage: model control group, MTX group, TPT group, and XFC group after 19th day. (1) TPT group: TPT was grinded into fine powder and mixed with saline to blending. The rats were intragastric administrated with 10 mL/kg 1 time each day. (2) MTX group: MTX was grinded into fine powder and mixed with saline to blending. The rats were intragastric administrated with 10 mL/kg 1 times a week, while they were given saline 10 mL/kg 1 times each day on the other days. (3) XFC group: XFC was grinded into fine powder without capsule and mixed with saline to blending. The rats were intragastric administrated with 10 mL/kg 1 times a day. (4) Normal control group and model control group were given saline 10 mL/kg 1 times each day. The rats of each group were intragastric administrated for 30 d.

### 2.5. The Voix Pedis' Swelling

Left voix pedis volumes of rats were measured on 1 d before injection, 1 d before administration, and 30 d after administration. The voix pedis' swelling was calculated [6]. The voix pedis' swelling = (*Vt* − *Vn*)/*Vn* × 100% (*Vn*, before injection; *Vt*, after injection).

### 2.6. Arthritis Index (AI)

Observe and record degree of arthropathy on 12th, 1 time three day. The arthropathy was appraised with 5-point method. AI was calculated according to the other three limbs without injection [7]. 0 score: no redness and swelling; 1 score: redness and swelling of little toe joint; 2 score: swelling of toe and voix pedis joint; 3 score: swelling of joints under ankle joint; 4 score: swelling of all joints includes ankle joint; 5 score: integral accumulation of each joint is AI of rats.

### 2.7. GMP-140

Blood was drawn to EDTA anticoagulant tube and then centrifuged for 8 minutes at 1000 r/min. Platelet rich plasma (PRP) is plasma without liquid supernatant. Then adding anti-rat GMP-140 monoclonal antibody labeled with FITC to it. And incubating for 15 minutes in dark at room temperature. Adding 2 mL 1% paraformaldehyde fixative and putting it to flow cytometry. Last, analyzing GMP-140 positive expression with CeQuest.

### 2.8. CD40L

Adding anti-rat CD40L monoclonal antibody labeled with FITC to 10 *μ*L blood with EDTA anticoagulant. And incubating for 5 minutes in dark at room temperature. Then centrifuging for 5 minutes at 1000 r/min. And throwing away liquid supernatant. Adding phosphate buffer (PBS) 2 mL and centrifuging for 5 minutes at 1000 r/min. Throwing away liquid supernatant. Mixing with PBS 300 *μ*L to blending. Putting it to flow cytometry and analyzing CD40L positive expression with CeQuest.

### 2.9. Statistics

Data were expressed as ($\bar{x}\pm s$) and to be treated statistically with SPSS 11.5 for windows software. After Kolmogorov-Smirnov Region Surrounding the Nansha Islands, multiple groups means were compared with single-factor analysis of variance, and the comparison among groups was performed with LSD and SNK method. And the samples were analyzed using the method of multivariate correlation analysis.

## 3. Result

### 3.1. The Change of Weight of Each Group

The weight of each group was no significant difference before phlegmasia (*P* > 0.05). The weight of model group was significantly decreased than the normal group on 1 day before administration (*P* < 0.05). Compared with the model group, the weight of MTX, TPT, and XFC groups was significantly increased (*P* < 0.01). The body mass in XFC-treated group was significantly higher than those in MTX and TPT groups (*P* < 0.05) (see Table 1).

### 3.2. The Change of the Voix Pedis' Swelling and AI of Each Group

The voix pedis' swelling and AI of the model group was significantly increased than the normal group on 1 day before administration (*P* < 0.01). Compared with the model group, there was no significant difference in three treated groups (*P* > 0.05). After the 30th of administration, the voix pedis' swelling and AI of MTX, TPT, and XFC groups was significantly decreased than those in the model group (*P* < 0.01). There was no significant difference among three treated groups (*P* > 0.05) (see Table 2). 

### 3.3. The Change of GMP-140, CD40L of Each Group

**Figure 1:**
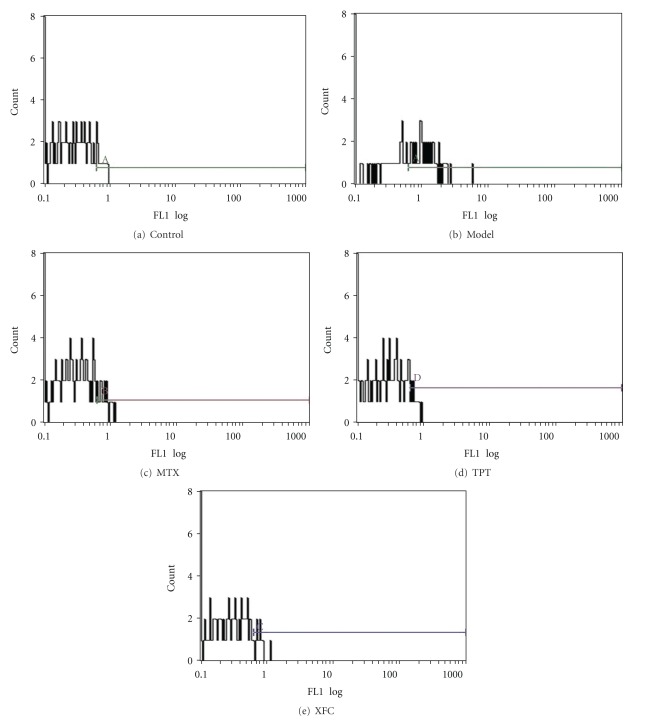
GMP-140 of AA rats.

**Figure 2:**
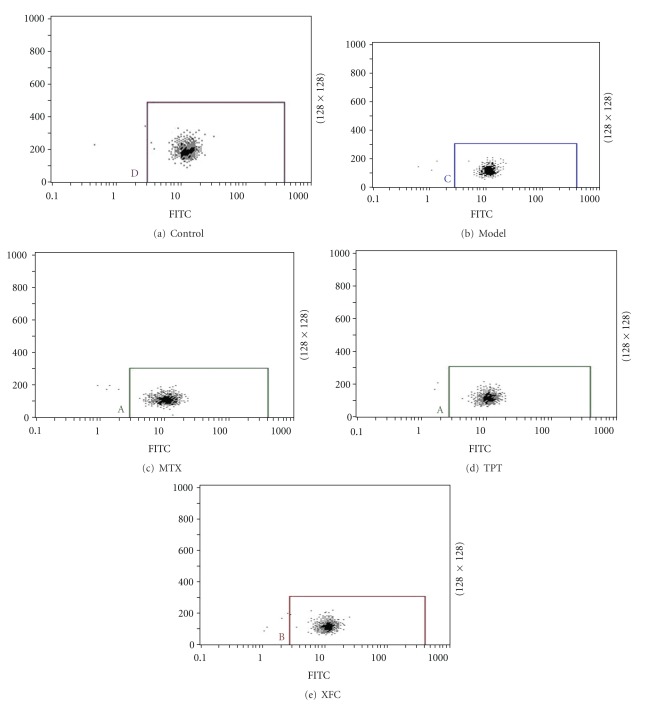
CD40L of AA rats.

The GMP-140, CD40L, of the model group was significantly higher than the normal group (*P* < 0.01). Compared with the model group, the GMP-140 and CD40L of MTX, TPT and XFC groups were significantly decreased (*P* < 0.01). The GMP-140 of XFC showed a decreasing tendency with no significant difference than the MTX, TPT groups (*P* > 0.05). The CD40L showed no significant difference among three treated groups (*P* > 0.05) (see Table 3 and Figures 1 and 2). 

### 3.4. The Dependability between GMP-140, CD40L, and the Voix Pedis' Swelling, AI of Rats

The expression of GMP-140 and CD40L were positively correlated with voix pedis' swelling and AI (*P* < 0.01 or *P* < 0.05) (see Table 4). 

## 4. Discussion

Platelet GMP-140 and CD40L as specific markers of platelet activation play an important role in the morbidity and development of rheumatoid arthritis [8]. Studies show that GMP-140 was the marker of RA activity and inflammatory [9, 10]. Activated GMP-140 interacted with polymorphonuclear leukocyte ligand in circulation. Then inflammatory cells were exuded to joint synovial tissues space and produced immune response and inflammatory damage of synovium. Inflammatory cells could release more GMP-140, thus, in cycles, promoting the progression of RA and synovial inflammatory [11]. GMP-140 could activate synovial blood coagulation system. Fibrin degradation products were deposited in RA synovium. It caused joint swelling and tissue injury [12]. CD40L plays an important role in the occurrence and development of chronic synovitis. High level expression of CD40L has been detected in RA synovium [13]. Expression of CD40L on platelet can induce endothelial cells to release chemotaxins and express adhesion molecules. Thus producing a signal, which raises various inflammatory mediators gathering in synovium. It causes hyperplasia of synovial tissue, inflammation, damage of cartilage and bone [14]. CD40L can also stimulate inflammation mononuclear macrophages, which secrete vascular endothelial growth factor, increase vascular permeability, improve inflammatory infiltration, and promote the growth and relocation of endothelial cells, angiogenesis, and pannus formation [15]. In this experiment, it was shown that GMP-140 and CD40L as platelet activation markers in peripheral blood of AA rats were significantly increased. The reason was that activated platelet in peripheral blood of AA rats releases a large number of activation products including platelet CD40L, GMP-140. This study also discovered that GMP-140 and CD40L were positively correlated with voix pedis' swelling and AI (*P* < 0.01 or *P* < 0.05). GMP-140 and CD40L as proinflammatory mediators promote the occurrence and development of joint synovitis and pannus formation. So, platelet GMP-140 and CD40L have relevance with inflammatory markers.

XFC, which strengthens the spleen and replenishes Qi, resolving Dampness and freeing channel, is a traditional Chinese medicine compound preparation based on overall regulation. Its main components are coix seed, astragalus, tripterygium wilfordii, and centipede. They not only have different degrees of anti-inflammatory effect but also inhibit platelet activation. Astragalus has obvious anti-inflammatory effects. Its mechanism could lie in decreasing the emergence of oxyradical, inflammatory mediators including IL-8, TNF-*α*, NO, and so on. And adding the expression of glucocorticoid receptor under inflammation [16, 17], astragalus can effectively reduce the degree of platelet activation and inhibit synthesis of platelet GMP-140. Modern study confirmed that it could obviously inhibit joint swelling induced by egg white, granuloma induced by cotton ball, and the ear edema in mice induced by xylene. And it also could inhibit inflammatory with inhibiting significantly the synthesis and release of prostaglandin E2 of inflammation [18]. Modern pharmacological studies show that tripterygium wilfordii had a strong anti-inflammation effect with inhibiting the expression of IL-1*β*, IL-12, TNF-*α*, and secretion of NO and IL-6 from macrophage [19–22]. Adjuvant arthritis rat blood was in hyperviscosity and platelet hyperaggregation. Tripterygium wilfordii could improve blood hyperviscosity and platelet aggregation of AA rat, which assisted anti-inflammatory effect. The research shows that tripterygium wilfordii could suppress immune by inhibiting expression of CD40L [23]. Centipede plays an anti-inflammatory effect with improving microcirculation, extending coagulation time, decreasing hematocrit, reducing blood viscosity, increasing the number of opening microvascular, and increasing diameter of microvascular.

The result from the experiment shows that XFC could decrease the voix pedis' swelling and AI of AA rats the same as MTX, TPT. The body mass in XFC-treated group was significantly higher than those in MTX and TPT groups because that astragalus in XFC could protect gastric mucosa and inhibit gastrointestinal adverse reactions of tripterygium wilfordii (*P* < 0.05). It shows that XFC has the anti-inflammatory effects as MTX, TPT, and is superior to MTX and TPT in improving overall function of AA rat. Content of tripterygium wilfordii in XFC is lower than TPT. It shows that XFC on the overall effect in AA rat is better than a separate application of TPT. And XFC could increase effect and inhibit toxic effect of tripterygium wilfordii. With the expression of GMP-140, CD40L in peripheral blood of AA rats decreased by XFC; the voix pedis' swelling and AI also dropped. It indicated that XFC could inhibit the inflammatory response through inhibiting platelet activation of AA rats, which means decreasing the expression of GMP-140, CD40L in peripheral blood. So, the voix pedis' swelling and AI were decreased as the result.

## Figures and Tables

**Table 1:** Comparison of weight of each group (*n* = 8, $\bar{x}\pm s$, g).

Groups	Weight		
	Weight before phlegmasia	Weight before administration	Weight of the 30th of administration
Control	214.38 ± 18.98	260.00 ± 11.02	360.25 ± 23.46
Model	211.88 ± 17.31	226.25 ± 35.73*	260.50 ± 37.20**
MTX	213.75 ± 13.02	231.88 ± 30.70*	311.00 ± 43.61^∗∆∆^
TPT	212.50 ± 11.95	230.00 ± 14.88*	317.63 ± 32.51^∗∆∆^
XFC	213.75 ± 22.80	232.50 ± 13.63*	351.88 ± 21.96^∆∆▲□^

Note: compared with control group, *P < 0.05, **P < 0.01; compared with model group, ^∆∆▲^ *P* < 0.01; compared with MTX group, ^▲^ *P* < 0.05; compared with TPT group, ^□^ *P* < 0.05.

**Table 2:** Comparison of the voix pedis' swelling, arthritis index of each group (*n* = 8,$\;\bar{x}\pm s$).

Groups	The voix pedis' swelling		AI	
	Weight before administration	Weight of the 30th of administration	Weight before administration	Weight of the 30th of administration
Control	33.28 ± 10.90	38.94 ± 24.69	0.00 ± 0.00	0.00 ± 0.00
Model	65.99 ± 16.49**	79.20 ± 24.59**	7.63 ± 0.92**	8.13 ± 0.64**
MTX	65.09 ± 14.11**	46.00 ± 14.94^∆∆^	7.25 ± 1.04**	4.00 ± 0.93^∗∗∆∆^
TPT	62.59 ± 20.19**	44.92 ± 20.37^∆∆^	7.13 ± 1.13**	3.88 ± 1.25^∗∗∆∆^
XFC	68.86 ± 16.41**	41.57 ± 15.28^∆∆^	7.88 ± 0.64**	3.50 ± 1.07^∗∗∆∆^

Note: compared with control group, **P < 0.01; compared with model group, ^∆∆^ *P* < 0.01.

**Table 3:** Comparison of GMP-140, CD40L of each group (*n* = 8, $\bar{x}\pm s$, pg/mL).

Groups	GMP-140	CD40L
Control	0.30 ± 0.17^∆∆^	94.71 ± 2.42^∆^
Model	1.07 ± 0.52**	98.16 ± 1.39**
MTX	0.55 ± 0.27^∆∆^	95.47 ± 2.36^∆^
TPT	0.49 ± 0.28^∆∆^	95.24 ± 1.90^∆^
XFC	0.38 ± 0.20^∆∆^	95.39 ± 2.27^∆^

Note: compared with control group, **P < 0.01; compared with model group, ^∆∆^ *P* < 0.01, ^∆^ *P* < 0.05.

**Table 4:** The dependability between GMP-140, CD40L, and the voix pedis' swelling, AI (*n* = 40).

	GMP-140		CD40L	
	*r*	*P*	*r*	*P*
The voix pedis' swelling	.608**	.000	.441*	.015
AI	.611**	.000	.386*	.035

**P < 0.01, *P < 0.05.
